# Role for endothelin-1 in cardiometabolic dysfunction with intermittent hypoxia

**DOI:** 10.1152/function.014.2026

**Published:** 2026-05-18

**Authors:** Anna M. Gonsalves, Sarah E. Baker, Prachi Singh, Jacqueline K. Limberg

**Affiliations:** ^1^Department of Nutrition and Exercise Physiology, University of Missouri, Columbia, Missouri, United States; ^2^Department of Anesthesiology Research, Mayo Clinic, Rochester, Minnesota, United States; ^3^Sleep and Cardiometabolic Health Lab, Pennington Biomedical Research Center, Louisiana State University, Baton Rouge, Louisiana, United States

**Keywords:** cardiometabolic disease, endothelin-1, intermittent hypoxia, sleep apnea, sympathetic nervous system

## Abstract

Endothelin-1 (ET-1) has been linked to increased hypoxic sensitivity of the chemoreceptors and the development of cardiometabolic disease. Repeated hypoxic exposures [i.e., intermittent hypoxia (IH)] elicit increases in ET-1, muscle sympathetic nerve activity (MSNA), and blood pressure (BP). In this brief review, we summarize data from our group(s) which examined the effect of acute IH on MSNA, BP, and indices of adipose tissue lipolysis [i.e., free fatty acids (FFAs)], as well as the role of ET-1 in these responses. Herein, we show that acute ET receptor inhibition (oral bosentan) attenuates the rise in BP during hypoxia and the fall in BP during hyperoxia in healthy young men. Although the effect of IH on MSNA and BP was not attenuated following ET-receptor inhibition, IH increased plasma ET-1, epinephrine, and FFA concentrations. Notably, inhibition of ET-1 receptors with oral bosentan attenuated the IH-mediated rise in epinephrine and FFA. With these findings, we demonstrate a role for ET-1 in the maintenance of resting BP in young men, possibly through a chemoreceptor-mediated mechanism. We further speculate that the effect of ET-1 receptor inhibition on circulating FFA is secondary to its ability to reduce sympathoadrenal tone during IH exposure. Results highlighted in this review may serve as the basis for future work exploring ET-1 as a therapeutic target for cardiometabolic dysfunction associated with IH.

## INTRODUCTION

Sleep apnea is the most prevalent form of sleep-disordered breathing, affecting 33% of adults in the United States (1). Patients with sleep apnea experience periodic cessations in breathing during sleep, defined clinically as ≥5 apneic or hypopneic events per hour of sleep (1). These recurrent events result in repeated bouts of arterial oxygen desaturation and hypoxemia, which rebound once breathing resumes (2). The pathophysiology of sleep apnea-associated cardiometabolic disease is multifactorial, including factors such as obesity, advanced age, and sleep fragmentation; however, a strong independent effect of intermittent hypoxic exposure [i.e., intermittent hypoxia (IH)] on cardiovascular and metabolic dysfunction is observed (3–5). In addition, the physiological response to IH includes profound activation of the sympathetic nervous system (6, 7), which further increases an individual’s risk of cardiometabolic disease (8–10).

**Figure 1.**
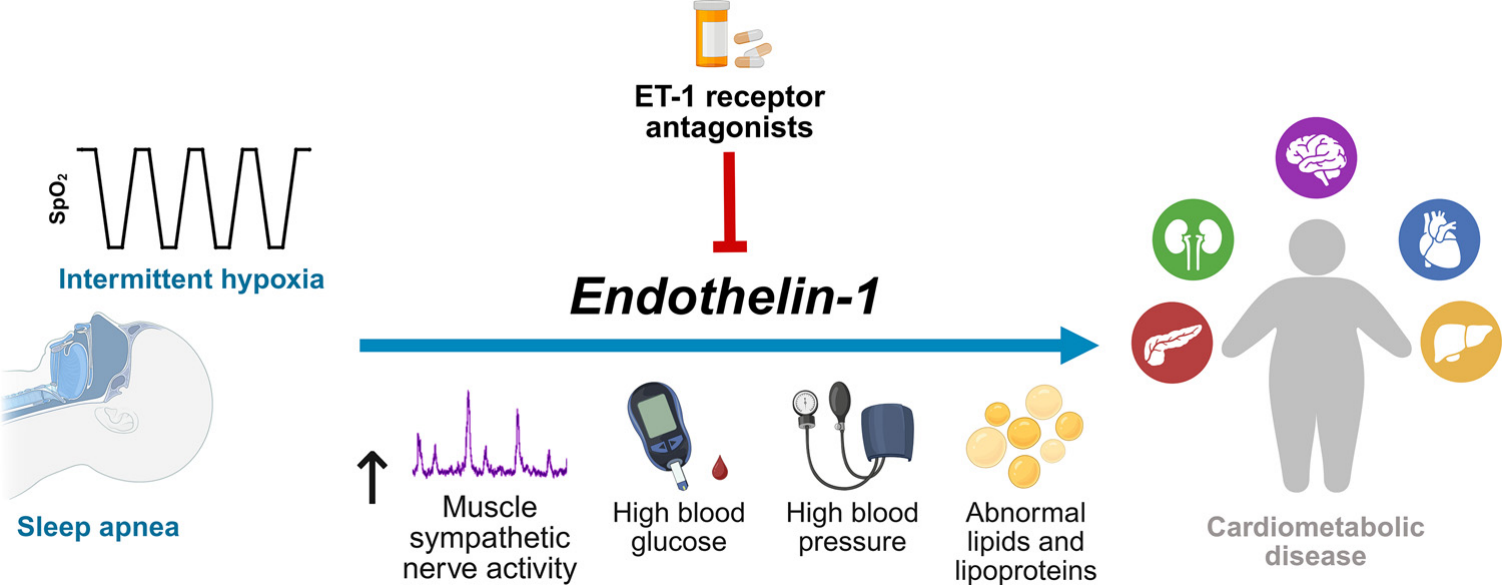
Herein, we present evidence from our group(s) implicating endothelin-1 (ET-1) as an integrative mediator linking the neural, vascular, and metabolic consequences of intermittent hypoxia (IH). Given the increasing prevalence of sleep apnea and its strong association with cardiometabolic disease, continued investigation into the role of ET-1 signaling is warranted to identify therapeutic opportunities that may mitigate cardiometabolic dysfunction in affected individuals.

Endothelin-1 (ET-1) has been linked to increased hypoxic sensitivity of chemoreceptors and the development of cardiometabolic disease in the context of IH (11–14). ET-1 is a potent vasoconstrictor peptide and the dominant effector of the endothelin system (15). ET-1 is produced in multiple tissues, including vascular endothelium, the central nervous system, and carotid body glomus cells (11, 14, 16), positioning it to integrate vascular and neural-chemoreflex control. In health, ET-1 contributes to the basal regulation of vascular tone through a balance of ET_A_-mediated vasoconstriction and ET_B_-mediated vasodilation (17). ET_A_ receptors are predominantly expressed on vascular smooth muscle cells, whereas ET_B_ are located on endothelial cells, where they promote nitric oxide- and prostacyclin-mediated vasodilation, as well as vascular smooth muscle cells, where they contribute to vasoconstriction. In this brief review, we synthesize current evidence on the role of ET-1 in mediating cardiovascular and metabolic responses to IH, with emphasis on data from our group(s) (Fig. 1).

## EFFECT OF INTERMITTENT HYPOXIA ON NEURAL CONTROL OF BLOOD PRESSURE

Patients with sleep apnea exhibit elevated sympathetic nervous system activity and risk of cardiovascular complications (7, 18–20). Transient exposures to low oxygen during sleep are a primary contributor to increases in sympathetic nervous system activity in adults with sleep apnea through repeated activation of the peripheral chemoreflex (7, 18, 21, 22). Increases in sympathetic nervous system activity promote neurotransmitter release (i.e., norepinephrine, epinephrine), which bind adrenergic receptors on the vascular smooth muscle, promoting vasoconstriction and increasing blood pressure (BP). Sympathetic outflow at the level of the peripheral vasculature can be measured directly from peripheral nerves in conscious humans via the technique of microneurography, termed muscle sympathetic nerve activity (MSNA) (23). Multiunit “bursts” of MSNA represent simultaneous firing of several axons near the tip of the microelectrode (24). Increases in MSNA are observed with exposure to IH in healthy humans, which persist beyond the period of hypoxia (25–33). Similarly, daytime MSNA is elevated in patients with sleep apnea, despite normal waking saturations (18). Elevated MSNA in patients with sleep apnea is achieved via increased firing probability and mean firing rates of individual neurons, as well as multiple within-burst firing (34, 35). Recent data from our group further support that, following acute IH, sympathetic neuronal discharge is increased in healthy humans via an increase in within-burst firing and recruitment of previously latent, larger amplitude action potentials (30). Both attenuation of carotid body chemoreceptor activity with acute hyperoxia (in healthy adults exposed to IH) (30) and continuous positive airway pressure (in patients with sleep apnea) (36, 37) can lower within-burst firing. Given that neural discharge determines neurotransmitter release (38) and ultimately promotes skeletal muscle vasoconstriction and increases in blood pressure (32, 39), these data support aberrant sympathetic neural firing in the pathogenesis of cardiovascular disease in individuals exposed to IH (e.g., sleep apnea).

## ROLE FOR ENDOTHELIN-1 IN NEURAL CONTROL OF BLOOD PRESSURE IN SETTING OF HYPOXIA

Numerous studies have linked ET-1 to hypoxic sensitivity of the chemoreceptors (11–14, 40–42), as well as neural control of blood pressure and the development of hypertension following IH (7, 43, 44). For example, patients with sleep apnea exhibit elevated plasma ET-1 levels (45–49), which are associated with the severity of nocturnal hypoxia (48, 49) and can be attenuated via continuous positive airway pressure treatment (48, 50). Plasma ET-1 levels are similarly increased following acute hypoxia in healthy men (51). Notably, the nonspecific ET-1 receptor antagonist, bosentan, blunts the blood pressure response to hypoxia in men with sleep apnea (52) and normalizes blood pressure regulatory mechanisms in rats exposed to IH (7, 44, 53). In addition, data suggest that bosentan may be as effective as continuous positive airway pressure in reducing clinic blood pressure in patients with sleep apnea (54).

With this information in mind, our laboratory tested whether short-term dual ET receptor antagonism with oral bosentan in healthy young men would *1*) lower resting blood pressure, *2*) blunt the blood pressure response to chemoreflex excitation with acute graded hypoxia, and *3*) attenuate the blood pressure fall during chemoreflex inhibition with transient hyperoxia (55). Our results demonstrated that short-term mixed ET_A_/ET_B_ receptor antagonism with bosentan lowers resting diastolic and mean arterial pressure in healthy young men, reinforcing the concept that endogenous ET-1 contributes to basal vascular tone even in the absence of overt cardiovascular disease (56). The absence of changes in systolic blood pressure or heart rate observed in our study underscores a predominantly vascular (vs. cardiac) mechanism, consistent with ET-1’s well-established actions on resistance vessels and its modest impact on cardiac output under physiological conditions. In addition, ET receptor blockade attenuated both the rise in mean blood pressure during hypoxia and the fall during hyperoxia, while leaving heart rate and ventilation largely unchanged. This pattern supports the preferential influence of ET-1 on the vascular arm of the chemoreflex, likely via modulation of sympathetic vasoconstrictor outflow or vascular responsiveness, rather than altering ventilatory drive or cardiac chronotropy. The preserved ventilatory and heart rate responses, combined with selective changes in mean blood pressure, are consistent with emerging concepts that distinct carotid body pathways and glomus cell populations can differentially regulate vascular and respiratory targets (57, 58). Collectively, these findings argue that ET-1 is an important contributor to both tonic and reflex support of blood pressure in healthy humans, operating at rest and during acute changes in chemoreceptor activity (55).

ET_A_ receptor antagonists can lower sympathetic nervous system activity and normalize blood pressure in male rats exposed to IH (7, 43, 44). To further assess this possibility in humans, we recruited a group of healthy young men to complete two 30-min sessions of IH, a control condition and following nonspecific ET-1 receptor blockade with oral bosentan (59). (In these initial studies, only men were recruited due to the teratogenic effects of bosentan.) Although we observed a significant increase in plasma ET-1 following acute IH exposure in healthy young men, which is consistent with data from others (51), ET-1 receptor antagonism did not prevent the MSNA- and blood pressure-raising effects of IH (59). Thus, despite a role for ET-1 in control of resting blood pressure in young men (55), we conclude ET-1 signaling is not obligatory for the neurovascular response to acute IH. With this, it is important to acknowledge that nonspecific ET_A_/ET_B_ receptor antagonism in our human study significantly restricts our ability to determine the potential varied effects of ET_A_- versus ET_B_-mediated effects and may contribute to the discrepancy from preclinical findings. Future studies in this area using receptor-specific antagonists are warranted.

## EFFECT OF INTERMITTENT HYPOXIA ON METABOLIC FUNCTION

In addition to an elevated risk for hypertension, sleep apnea increases an individual’s risk of developing insulin resistance and type 2 diabetes, independent of comorbidities such as obesity (60–65). Indeed, fasting hyperglycemia is consistently reported following acute exposure to hypoxia in both mice and humans (66, 67). Although mechanistic understanding is incomplete, IH has been shown to augment glucose output from the liver via increases in sympathetic nervous system activity (68). Exposure to IH further contributes to impairments in glucose metabolism, including decreased insulin sensitivity, glucose effectiveness, and insulin secretion (66, 69–74). Notably, treatment with continuous positive airway pressure in patients with sleep apnea (75) or supplemental oxygen in patients with chronic obstructive pulmonary disease (76) can reduce insulin resistance, supporting an important role for hypoxia in this response.

To address such questions in humans, we conducted a randomized, single-blinded, crossover study in healthy young adults, which uncovered an immediate (within 30 min) and prolonged (up to 180 min) increase in circulating glucose concentrations with IH exposure (77). Despite limited effects on insulin secretion and/or sensitivity following acute IH in healthy young adults, our study uncovered that the amount of insulin the liver allowed into the systemic circulation was attenuated (77). Results agree with data showing little effect of hypoxia on insulin sensitivity and glucose tolerance, but altered insulin clearance (78). It is also reasonable to propose that acute increases in plasma glucose following IH may be the result of an increase in adipose tissue lipolysis, an idea supported in the preclinical literature (79). Free fatty acid (FFA) levels are similarly elevated in patients with sleep apnea (80–82), and the rise in FFA is abolished with supplemental oxygen (82). Given that over 70% of adults with type 2 diabetes have concurrent sleep apnea (61, 83–86) and up to 30% of adults with sleep apnea have type 2 diabetes (60, 87–90), these data may have implications for altered glucose handling observed in this population and warrant additional mechanistic research to identify potential therapeutic targets.

## ROLE FOR ENDOTHELIN-1 IN METABOLIC DYSFUNCTION ASSOCIATED WITH INTERMITTENT HYPOXIA

Elevated plasma ET-1 has been reported in individuals with insulin resistance with and without sleep apnea (49, 91, 92). Using an in vitro approach, we demonstrated that IH directly mediates vascular insulin resistance via an imbalance in insulin-mediated nitric oxide and ET-1 expression and secretion (93). ET-1 receptors are additionally expressed on adipocytes and have been proposed to contribute to the activation of adipose tissue lipolysis (94–97). Lipolysis increases circulating FFA, and experimental elevations in FFA have been shown to induce insulin resistance in healthy adults (98). Accordingly, sleep apnea-associated increases in ET-1 may represent a mechanistic link between elevated FFA and metabolic dysfunction. Indeed, IH increases ET-1 secretion and ET-1 receptor activation in adipose tissue in rodent models (95).

Using a crossover study design, we examined the effects of IH on adipose tissue lipolysis and evaluated the potential contribution of ET-1 to this response (99). Consistent with our hypothesis, plasma FFA responses to IH were attenuated during ET-receptor blockade with bosentan compared with control conditions in healthy young men. However, experiments from abdominal subcutaneous adipose tissue biopsies found no effect of acute bosentan on transcription of cellular receptors/proteins. Additional in vivo experiments in primary differentiated human white preadipocytes demonstrated that ET-1 did not exert direct lipolytic effects. These data led us to speculate that bosentan-mediated attenuation of lipolysis was unlikely to have occurred via direct ET-1 receptor signaling at the level of the adipocyte, but rather through upstream lipolytic processes. In support of this idea, we observed lower circulating epinephrine concentrations following bosentan treatment and an attenuated epinephrine response to IH (99). Based on these findings, we propose that bosentan attenuates adrenomedullary tone and, by extension, epinephrine-mediated induction of lipolysis during IH and aligns with our understanding of the contribution of sympathetic activation to sleep apnea pathophysiology (7, 18, 21, 22). We further speculate that chronic ET-receptor antagonism may blunt transient increases in circulating FFA in patients with sleep apnea and expand the role of ET-1 in mediating metabolic dysfunction associated with IH (95, 100); however, to our knowledge, this idea remains untested in humans. Better characterization of ET-1 receptor expression across freshly isolated visceral (vs. subcutaneous) adipocytes and primary cell lines is warranted to address this important question.

It is also important to note that the abovementioned research was conducted in normal weight, insulin-sensitive men, and both the IH exposure (30 min) and oral bosentan treatments (3 days) were relatively acute. Thus, our results do not address metabolic responses in obesity or in chronic inflammatory conditions and insulin resistance often observed in adults with sleep apnea, where ET-1 signaling may play a more prominent role (101, 102). Indeed, high-fat diet-fed mice treated with either ET_A_ receptor blockade or dual ET_A_/_B_ receptor blockade demonstrate improved glucose handling, insulin sensitivity, dyslipidemia, adipokine levels, and epididymal white adipose tissue (eWAT) inflammation, independent of changes in body weight or body composition (103). In addition, dual ET-receptor antagonism improves HOMA-IR and glucose tolerance in mice with systemic lupus erythematosus, an autoimmune disease associated with obesity, insulin resistance, and elevated ET-1 (104). Relevant to the current model, in mice exposed to IH, selective ET_B_ receptor blockade (with BQ-788) but not ET_A_ blockade (with BQ-123) ameliorated IH-induced glucose intolerance and prevented worsening of insulin resistance, suggesting a role for ET-1 signaling in IH-associated glucose dysregulation and highlighting potential receptor-specific dynamics with IH (100). Future studies in patients with sleep apnea, as well as the application of receptor-specific antagonists, are warranted to test whether chronic ET-receptor antagonism improves metabolic outcomes and translates preclinical findings within the context of IH.

## POTENTIAL SEX-RELATED DIFFERENCES

In all our prior work examining a role for ET-1 in cardiometabolic dysfunction in response to IH exposure in humans, only men were studied. This decision was made due to the teratogenic nature of the study drug (bosentan). With this, it is important to acknowledge that the generalization of results to women is not likely. Unfortunately, data from women are limited due to their relative underrepresentation in research, as well as underdiagnosis (105) and undertreatment (106) of women with sleep apnea. Given the relatively homogeneous study population (young, lean, otherwise healthy men) in the majority of research presented herein, further investigation in more diverse populations, including consideration of sex-specific differences, is warranted. This is particularly relevant considering known sex-related differences in vascular (107) and adipose (108) ET-1 signaling.

Very little is known regarding sex differences in the neural response to IH; however, data from our group (29) and others (109–111) refute the likelihood of sex-related differences in the MSNA response to IH. In contrast to a lack of sex-related differences in the MSNA response to IH, we (29) and others (112, 113) have observed an attenuated blood pressure response to IH in women, which aligns with a lower incidence of hypertension in women with sleep apnea compared with men (114–116). Emerging evidence suggests that ET receptor-specific mechanisms may differ by sex, with women demonstrating greater ET_B_ receptor-mediated vasodilatory influence and men exhibiting greater reliance on ET_A_-mediated vasoconstriction (117). ET receptor responses are modulated by ovarian hormones in women, such that ET_B_ receptor-mediated vasodilation is greater in the presence of estrogen (118, 119), and elevations in endogenous ovarian hormones attenuate the vasoconstrictor capacity of ET_A_ (119). Furthermore, the vasodilatory capacity and endothelial expression of the ET_B_ receptor are reduced following menopause, suggesting a potential mechanism linking hormonal status to vascular dysfunction with aging (118, 120). Although these findings support the notion of sex- and hormone-dependent differences in ET receptor subtype signaling, whether these differences contribute to the divergent responses to IH has yet to be investigated.

## CLOSING REMARKS

Present evidence implicates ET-1 as an integrative mediator linking the neural, vascular, and metabolic consequences of IH (Fig. 1). Given the increasing prevalence of sleep apnea and its strong association with cardiometabolic disease (Fig. 1), continued investigation into the role of ET-1 signaling is warranted to identify therapeutic opportunities that may mitigate cardiometabolic dysfunction in affected individuals.
